# Coherent population transfer between uncoupled or weakly coupled states in ladder-type superconducting qutrits

**DOI:** 10.1038/ncomms11018

**Published:** 2016-03-24

**Authors:** H. K. Xu, C. Song, W. Y. Liu, G. M. Xue, F. F. Su, H. Deng, Ye Tian, D. N. Zheng, Siyuan Han, Y. P. Zhong, H. Wang, Yu-xi Liu, S. P. Zhao

**Affiliations:** 1 Beijing National Laboratory for Condensed Matter Physics, Institute of Physics, Chinese Academy of Sciences, Beijing 100190, China; 2 Department of Physics, Zhejiang University, Hangzhou 310027, China; 3 Department of Physics and Astronomy, University of Kansas, Lawrence, Kansas 66045, USA; 4 Institute of Microelectronics, Tsinghua University, Beijing 100084, China; 5 Tsinghua National Laboratory for Information Science and Technology (TNList), Beijing 100084, China; 6 Collaborative Innovation Center of Quantum Matter, Beijing, China

## Abstract

Stimulated Raman adiabatic passage offers significant advantages for coherent population transfer between uncoupled or weakly coupled states and has the potential of realizing efficient quantum gate, qubit entanglement and quantum information transfer. Here we report on the realization of the process in the superconducting Xmon and phase qutrits—two ladder-type three-level systems in which the ground state population is coherently transferred to the second excited state via the dark state subspace. We demonstrate that the population transfer efficiency is no less than 96% and 67% for the two devices, which agree well with the numerical simulation of the master equation. Population transfer via stimulated Raman adiabatic passage is significantly more robust against variations of the experimental parameters compared with that via the conventional resonant π pulse method. Our work opens up a new venue for exploring the process for quantum information processing using the superconducting artificial atoms.

## Abstract (web summary)

Quantum state engineering necessitates transfer between quantum states. Here the authors demonstrate coherent population transfer between un- or weakly-coupled states of solid state systems, superconducting Xmon and phase qutrits, using stimulated Raman adiabatic passage and microwave driving.

## Graphical abstract

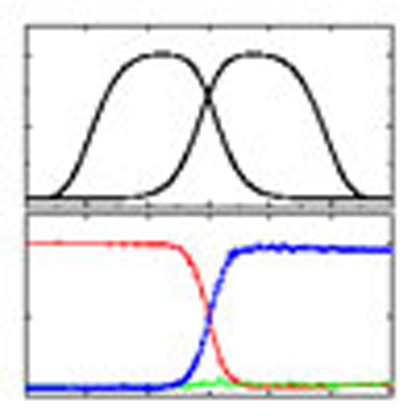

Stimulated Raman adiabatic passage (STIRAP), which combines the processes of stimulated Raman scattering and dark state adiabatic passage, is a powerful tool used for coherent population transfer (CPT) between uncoupled or weakly coupled quantum states^1^,^2^,^3^. It has been recognized as an important technique in quantum computing and circuit quantum electrodynamics involving superconducting qubits^4^,^5^,^6^,^7^,^8^,^9^,^10^,^11^,^12^,^13^. For example, qubit rotations can be realized via STIRAP with two computational states plus an auxiliary state forming a three-level Λ configuration^4^,^5^. A scheme for generating arbitrary rotation and entanglement in the three-level Λ-type flux qutrits is also proposed^6^, and the experimental feasibility of realizing quantum information transfer and entanglement between qubits inside microwave cavities has been discussed^7^,^8^. Unlike the conventional resonant π pulse method STIRAP is known to be much more robust against variations in experimental parameters, such as the frequency, amplitude and interaction time of microwave fields and the environmental noise^5^,^6^,^11^,^12^.

Recently, multi-level systems (qutrits or qudits) have found important applications in speeding up quantum gates^14^, realizing quantum algorithms^15^, simulating quantum systems consisting of spins greater than one half^16^, implementing full quantum-state tomography^17^,^18^,^19^, testing quantum contextuality^20^ and mapping to multi-qubit systems^21^,^22^. Unlike the highly anharmonic Λ-type flux qutrits the phase and transmon (or Xmon) qutrits have the ladder-type (Ξ-type) three-level configuration which is weakly anharmonic. The dipole coupling between the ground state |0〉 and the second excited state |2〉 in the phase qutrit is much weaker than those between the first excited state |1〉 and the |0〉 state or the |2〉 state. In the case of the transmon (or Xmon) qutrit the dipole coupling is simply zero. This unique property makes it difficult to transfer population from |0〉 to |2〉 directly using a single π pulse tuned to their level spacing *ω*_20_. The usual solution is to use the high-power resonant two-photon process or to apply two successive π pulses transferring the population first from |0〉 to |1〉 and then from |1〉 to |2〉 (refs 18, 19). These methods often lead to a significant population in the middle level |1〉 resulting in energy relaxation which degrades the transfer process. In contrast, STIRAP transfers the qutrit population directly from |0〉 to |2〉 via the dark state subspace without occupying the middle level |1〉.

In this work, we report on the realization of STIRAP in the Ξ-type superconducting Xmon^23^ and phase^24^ qutrits. We demonstrate CPT from the ground state |0〉 to the second excited state |2〉 via STIRAP in the Xmon and phase qutrits in which population transfer efficiency no less than 96% and 67% is achieved, respectively. The experimental results are well described by the numerical simulation of the master equation.

## Results

### The STIRAP concept

**Figure 1:**
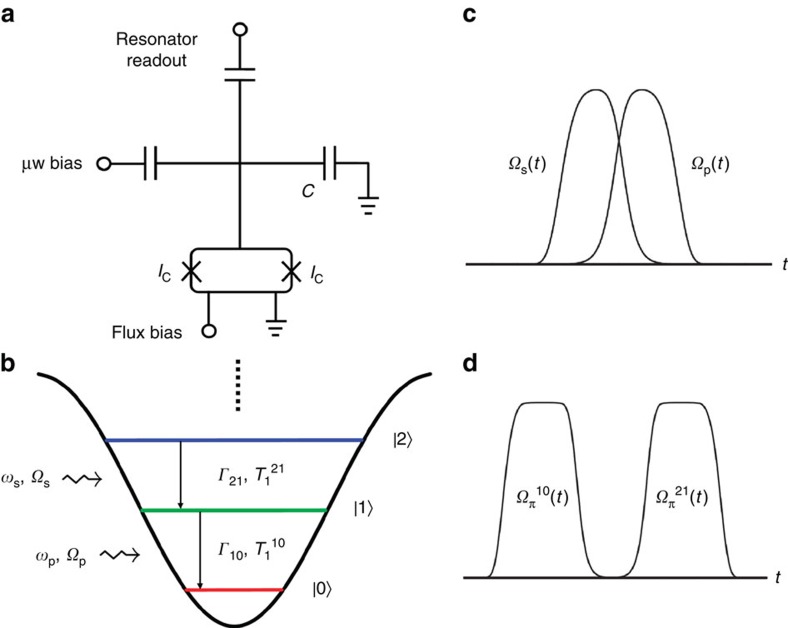
Superconducting Xmon qutrit and measurement pulse sequences. (**a**) Schematic Xmon qutrit with Josephson critical current *I*_c_ and shunt capacitance *C*. (**b**) Three bottom energy levels |0〉, |1〉 and |2〉 of the qutrit with related symbols indicated. Subscripts p and s refer to the pump and Stokes tones, respectively. (**c**) Counterintuitive pulse sequence with Ω_s_ preceding Ω_p_ for coherent population transfer from |0〉 to |2〉 without involving |1〉. (**d**) Conventional resonant π pulse sequence for successive |0〉→|1〉→|2〉 population transfers.

For clarity, our results will be mainly presented for the Xmon qutrit, which has longer coherence times and thus better performance, while those for the phase qutrit will be discussed as a comparison showing the effect of system decoherence. As is shown schematically in Fig. 1a, the Xmon qutrit has a shunt capacitance *C* and two Josephson junctions each with critical current *I*_c_ to form a SQUID loop so the potential and level spacing can be tuned via the flux bias. The potential energy and quantized levels |0〉, |1〉 and |2〉 of the qutrit are illustrated in Fig. 1b in which the frequencies *ω*_p,s_ of the pump and Stokes fields and their strength Ω_p,s_ (Rabi frequencies) are also indicated. Since the matrix element between the |1〉 and |2〉 states is a factor of 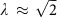 larger than that between the |0〉 and |1〉 states for both the Xmon and phase qutrits with weak anharmonicity^25^,^26^,^27^, applying the rotating-wave approximation in the double-rotating frame the Hamiltonian can be written as^26^,^27^:


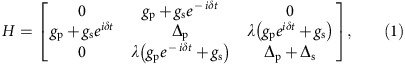


where the Planck constant *ħ* is set to unity, *δ*=*ω*_p_−*ω*_s_, Δ_p_=*ω*_10_−*ω*_p_ and Δ_s_=*ω*_21_−*ω*_s_ are various detunings, *g*_p,s_ are the qutrit microwave couplings proportional to the amplitudes of the pump and Stokes fields, respectively. In equation (1), the matrix element between the |0〉 and |2〉 states is zero, which is true for the Xmon and is a good approximation for the phase qutrit^27^. Hence, the Hamiltonian can be used to describe both devices. For 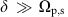 the fast-oscillating terms in the equation averages out to zero so the Hamiltonian becomes


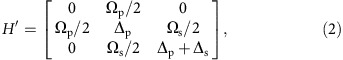


in which Ω_p_=2*g*_p_ and Ω_s_=2*λg*_s_. Equation (2) is the well-known rotating-wave approximation Raman Hamiltonian^1^,^2^. In particular, when the system satisfies the pump and Stokes two-photon resonant condition:


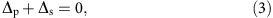


it has an eigenstate |*D*〉=cos Θ|0〉−sin Θ|2〉, called the dark state, which corresponds to the eigenvalue of 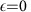. Here tan Θ(*t*)=Ω_p_(*t*)/Ω_s_(*t*). CPT from the ground state |0〉 to the second excited state |2〉 without populating the first excited state |1〉 can therefore be realized via STIRAP by initializing the qutrit in the ground state |0〉 (refs 27, 28), and then slowly increasing the ratio Ω_p_(*t*)/Ω_s_(*t*) to infinity as long as the following conditions^1^,^2^,^29^,^30^


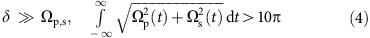


are satisfied so that the qutrit will stay in the dark state subspace spanned by {|0〉, |2〉}. The first condition is required to reduce equation (1) to equation (2) leading to the existence of the dark state solution, while the second ensures the adiabatic state following.

### Sample parameters and measurements

The Xmon qutrit used in this work is an aluminum-based device^23^, which is cooled down to *T*≈10 mK in the cryogen-free dilution refrigerator. A dispersive readout scheme with additional gains from a parametric amplifier is used to detect the qutrit states (see Methods). For the present experiment, the lowest three levels used as the qutrit states have the relevant transition frequencies of *f*_10_=*ω*_10_/2π=6.101 GHz and *f*_21_=*ω*_21_/2π=5.874 GHz, and the relative anharmonicity is *α*=(*f*_10_−*f*_21_)/*f*_10_≈3.7%. The measured energy relaxation times are 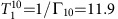 μs and 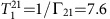 μs, respectively, while the dephasing time determined from Ramsey interference experiment is 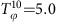 μs. To realize STIRAP, a pair of bell-shaped counterintuitive microwave pulses with the Stokes pulse preceding the pump pulse, as illustrated in Fig. 1c, are used. The pulses are defined by Ω_s_(*t*)=Ω_0_*F*(*t*) cos[π*η*(*t*)/2] and Ω_p_(*t*)=Ω_0_*F*(*t*)sin[π*η*(*t*)/2] with 
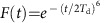
 and 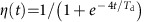, respectively^2^,^30^.

### Coherent population transfer

**Figure 2:**
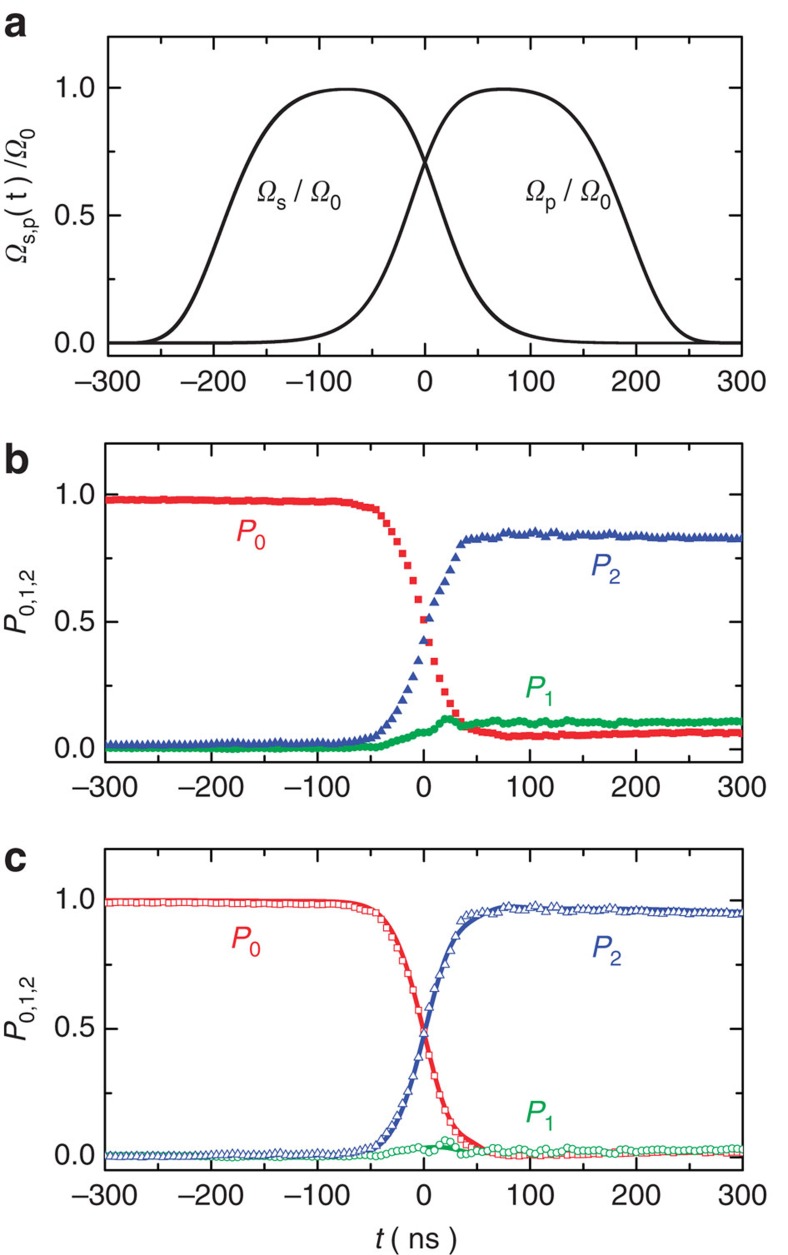
Coherent population transfer via STIRAP in the superconducting Xmon qutrit. (**a**) Stokes and pump microwave pulses Ω_s_(*t*) and Ω_p_(*t*) with the experimental parameters *ω*_s_/2π=*f*_21_=5.874 GHz, *ω*_p_/2π=*f*_10_=6.101 GHz, *Ω*_0_/2π=30 MHz and *T*_d_=100 ns. (**b**) Measured level populations *P*_0_, *P*_1_ and *P*_2_ versus time with a maximum value of *P*_2_=85% driven by the STIRAP pulse pair in **a** in the case of Δ_p_=Δ_s_=0. (**c**) Experimental level populations with maximum *P*_2_ reaching 97% (symbols) after correcting the readout imperfection as described in Methods. The lines are the numerical results calculated using the master equation considering the relaxation and dephasing processes, which agree well with the experimental data after correction. The experimentally determined parameters are used in the calculation: Γ_10_=8.4 × 10^4^ s^−1^, Γ_21_=1.3 × 10^5^ s^−1^, and 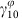
=2.0 × 10^5^ s^−1^. Other parameters in the master equation are taken as 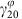
≈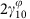 and 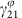

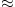

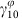
.

Figure 2a shows the two microwave pulses defined by Ω_0_/2π=30 MHz and *T*_d_=100 ns. As *t* increases, Ω_s_(*t*) and Ω_p_(*t*) start to increase and decrease, respectively, across *t*=0 at which they are equal. The experimentally measured populations *P*_0_, *P*_1_, and *P*_2_ versus time produced by this counterintuitive pulse sequence in the resonant case Δ_p_=Δ_s_=0 are plotted in Fig. 2b. We observe that as time evolves across *t*=0 the population *P*_2_ (*P*_0_) increases (decreases) rapidly while *P*_1_ remains low, signifying the occurrence of STIRAP via the dark state of the superconducting qutrit system. The experimentally measured maximum *P*_2_ is about 85% for the present sample under the resonant condition. The maximum value of *P*_2_ can be defined as the transfer efficiency or fidelity of the STIRAP process. As discussed in Supplementary Note 1, the experimentally measured value is much limited by the state preparation and measurement (SPAM) errors^31^ for the Xmon qutrit. In Fig. 2c, we show the corrected experimental data (symbols) assuming that SPAM errors are mostly due to the readout imperfection (see Methods section). The transfer efficiency after correction reaches 97% and the results match very well with the numerical simulations shown in the figure as solid lines. To further check the influence of the state preparation error ignored in the readout correction, we carry out a series of rigorous calibrations using the standard randomized benchmarking (Supplementary Fig. 1), sequential double π pulses (Supplementary Figs 2 and 3), and sequential STIRAP double π pulses (Supplementary Fig. 4) methods and demonstrate that the transfer efficiency is no less than 96%, which is close to the value after readout correction indicating that the influence of the state preparation error is negligible. The calibrations are detailed in Supplementary Note 1.

Notice that in the entire region of *t*∈[−300, 300] ns, all of the characteristic features of the experimental data, in particular (i) *P*_1_ remaining significantly lower than *P*_2_, (ii) the slight decrease (increase) of *P*_2_ (*P*_0_) after reaching the maximum (minimum) as well as the slight rising of *P*_1_, are reproduced well by the numerical simulations. The simulated temporal profiles of the populations *P*_0_, *P*_1_, and *P*_2_ are obtained by solving the master equation 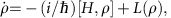 using the measured qutrit parameters, where *L*(*ρ*) is the Liouvillean containing the relaxation and dephasing processes^27^ (see Methods section). The numerical results also show that feature (ii) is due primarily to energy relaxation, while the maximum *P*_2_ reachable would mainly be limited by dephasing, which can be seen more clearly for the phase qutrit (Supplementary Fig. 5) having shorter coherence times as presented and discussed in Supplementary Note 2.

In our experiment the conditions imposed by equation (4) are satisfied: *δ*/2π in the resonant case Δ_p_=Δ_s_=0 is *f*_10_−*f*_21_=227 MHz, which is ∼7.5 times that of Ω_0_/2π, and it is easy to verify that the integrated pulse area 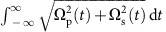
≈22π is greater than 10π. We point out that in addition to the influence of coherence times, the transfer efficiency of the demonstrated STIRAP process can also be improved by increasing the relatively small anharmonicity parameter *α*≈3.7% of the present sample up to, for example, 10% by optimizing device parameters of the Ξ-type phase^32^ and transmon (or Xmon)^33^ qutrits. According to equation (4) larger anharmonicity allows the use of larger *Ω*_0_ which would proportionally reduce the duration of the pump and Stokes pulses when the pulse area is kept unchanged to satisfy the adiabatic condition. Shorter pulses also reduce the negative effect of decoherence on the transfer efficiency^3^,^13^.

### Bright and dark resonances

**Figure 3:**
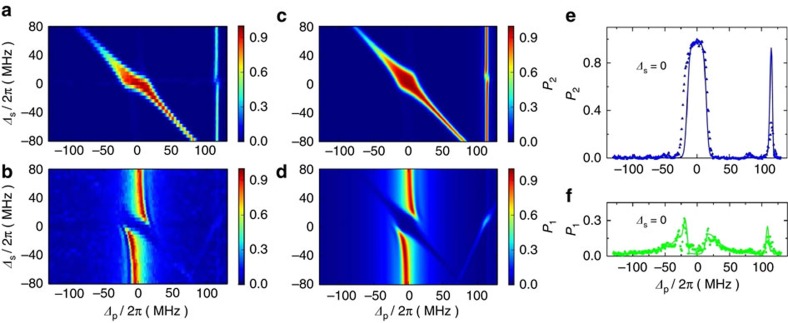
Bright and dark resonances. Level populations *P*_2_ and *P*_1_ taken at *t*=100 ns versus detunings Δ_s_ and Δ_p_. (**a**,**b**) Experimental; (**c**,**d**) theoretical. Bright and dark resonances can be seen clearly in **a**–**d**, respectively. The right-side peaks in **a**,**c** result from the two-photon process of the single pump microwave tone. (**e**) Bright and (**f**) dark resonance data plotted with Δ_s_=0. Symbols and lines are, respectively, the experimental results and the results calculated using the same parameters in Fig. 2.

The STIRAP process is often identified in either the time domain or the frequency domain^1^,^2^. The latter is based on equation (3) which specifies the pump and Stokes two-photon resonance condition. In Fig. 3a,b, we show the corrected experimental level populations *P*_2_ and *P*_1_ under the variations of the pump and Stokes detunings Δ_p_ and Δ_s_, respectively. The results are accompanied by the numerical simulations via the master equation (Fig. 3c,d) with fair agreement. Bright and dark resonances can be seen clearly in Fig. 3a,c and Fig. 3b,d, respectively. The bright resonance manifests itself as a stretched line with large *P*_2_ from the top-left to bottom-right corners reflecting the resonance condition equation (3), and with a much extended area near Δ_s_, Δ_p_∼0. The dark resonance appears as small *P*_1_ in areas wherever *P*_2_ is large. The other two highly populated areas can also be seen. One is *P*_2_ excited by the two-photon process from the single pump microwave tone, appearing as a thin vertical line on the right side in Fig. 3a,c. A split of the line near Δ_s_=0 can be seen, which could result from the Autler–Townes splitting of the |2〉 level induced by the Stokes microwave tone. The other is the vertical stripes near Δ_p_=0 in Fig. 3b,d originating from the resonant excitation of *P*_1_ by the pump microwave tone. However, the stripes are distorted near Δ_s_=0 due to the dark resonance from the STIRAP process.

In Fig. 3e,f, we compare the populations of the bright (*P*_2_) and dark (*P*_1_) states as a function of pump field detuning Δ_p_ when the frequency of the Stokes field resonates with *ω*_21_/2π (that is, Δ_s_=0). While the agreement between the measured and simulated *P*_1_ is pretty well those of *P*_2_ differ significantly in the height of the right-side peak around Δ_p_=115 MHz that results from the single pump tone two-photon process. At present, it is not clear what is the cause for this discrepancy. However, because the two-photon resonance is located far away from the intended parameter region of STIRAP its effect on the efficiency and robustness of the coherent population transfer can be ignored.

### Uniqueness and robustness

Similar results are obtained for the phase qutrit (Supplementary Figs 6 and 7) with a relative anharmonicity of *α*=2.9% and shorter coherence times on the order of a few hundred nanoseconds, in which a coherent population transfer efficiency as high as 67% is achieved, consistent with the numerical simulations using the experimentally determined sample parameters listed in Supplementary Table 1 (see discussions in Supplementary Note 2). All these results demonstrate clearly CPT from the ground state |0〉 to the second excited state |2〉 via STIRAP in the Ξ-type superconducting qutrits. We note that compared with the usual high-power single-tone two-photon process or two non-overlapping successive resonant *π* pulse excitations shown in Fig. 1d, which involve significant undesired population in the middle level |1〉 and require precise single photon resonance and pulse area^11^,^18^, CPT via STIRAP demonstrates simply the opposite. First, in principle CPT between |0〉 and |2〉 can be accomplished without occupying the lossy middle level |1〉. More importantly, the process is much more robust against variations in the frequency, duration and shape of the driving pulses^1^,^2^. In fact, in terms of equation (3) and equation (4), we see from Fig. 3a,c,e that the pump and Stokes tones resonance condition is greatly relaxed due to a much wider peak width of the STIRAP process as compared, for example, with the single-tone two-photon excitation from |0〉 state to |2〉 state having a much narrow peak. On the other hand, although Ω_p,s_ are limited by the system anharmonicity, their values, together with *T*_d_, still have much room for variations while maintaining the transfer efficiency. Our simulated results indicate that the transfer efficiency of STIRAP is very insensitive to Ω_0_, which is limited by systems anharmonicity, and to *T*_d_, which should be much smaller than the coherence time. The allowed variations for the present Xmon qutrit are about 20 MHz in Ω_0_ and 100 ns in *T*_d_ for keeping *P*_2_≥96%, which are in sharp contrast to, for example, the case of simple *π* pulse excitations. The extreme robustness of the STIRAP process is very advantageous and should be useful in various applications such as realizing efficient qubit rotation, entanglement and quantum information transfer in various superconducting qubit and qutrit systems.

## Discussion

We have experimentally demonstrated coherent population transfer between two uncoupled or weakly coupled states, |0〉 and |2〉, of the superconducting Xmon and phase qutrits having Ξ-type ladder configuration via STIRAP. The qutrits had small relative anharmonicity around 3% and moderate coherence times ranging from a few hundred ns up to ten μs. We demonstrated that by applying a pair of counterintuitive microwave pulses in which the Stokes tone precedes the pump tone, coherent population transfer from |0〉 to |2〉 with efficiency no less than 96% and 67% for the two devices can be achieved with a much smaller population in the first excited state |1〉. Using the measured qutrit parameters, including coherence times, we simulated the STIRAP process by numerically solving the master equation. The results agreed well with the experimental data.

Coherent population transfer via STIRAP is much more robust against variations of the experimental parameters, including the amplitude, detuning and time duration of the microwave fields, and the environmental noise over the conventional methods such as using high-power single-tone two-photon excitation and two resonant π pulses tuned to *ω*_10_ and *ω*_21_, respectively. Therefore STIRAP is advantageous for achieving robust coherent population transfer in the ladder-type superconducting artificial atoms that play increasingly important roles in various fields ranging from fundamental physics to quantum information processing. With improved qutrit parameters of coherence times up to 40 μs, presently attainable in the Xmon^23^, transmon^33^,^34^ and flux^35^ type devices, nearly complete transfer above 99% from level |0〉 to level |2〉 while keeping the level |1〉 population below 1% is expected. On the other hand, STIRAP in the Λ-type systems^3^ such as superconducting flux qutrits, in which the initial and target states locate in different potential wells representing circulating currents in opposite directions, is important in various applications and its experimental implementation remains to be explored. Our work paves the way for further progress in these directions.

## Method

### Dispersive readout of Xmon qutrit and SPAM errors

The Xmon qutrit is capacitively coupled to an on-chip *λ*/4 coplanar waveguide resonator which has a fixed resonant frequency at *ω*_r_/2π≈6.640 GHz. The qutrit-resonator coupling strength is designed to be about 30 MHz if on-resonance, and the coplanar waveguide resonator is loaded to external circuitry whose microwave response can be probed in terms of its transmission coefficient *S*. As the Xmon qutrit is far detuned from *ω*_r_, there is a dispersion-induced resonant frequency shift of the resonator, that is, the resulting transmission coefficient *S* expressed by a complex number *I*+*iQ* takes different values depending on the exact qutrit state. For readout we input an 800-ns-long microwave pulse, which is ∼1 MHz detuned from *ω*_r_/2π, and the output microwave pulse with the desired resonator information encoded in (*I*, *Q*) is sequentially amplified at multiple stages using a Josephson junction parametric amplifier^36^ and other low-noise amplifiers before demodulated by room temperature electronics^37^.

In the perfect absence of noise we would obtain three signal points in the *I*–*Q* plane for the qutrit's three eigenstates |0〉, |1〉 and |2〉, respectively. However, unavoidable noise in the measurement system gives rise to random scattering of the signal points around the ideal values, resulting in effectively three circular clouds corresponding to the three eigenstates. For a single measurement event in which a point (*I*, *Q*) is demodulated from an 800-ns-long microwave pulse, we categorize the qutrit state according to the minimum distance between this point (*I*, *Q*) and the three cloud centres. We repeat the sequence several hundred or thousand times for many points of (*I*, *Q*)s, from which the occupation probabilities for |0〉, |1〉 and |2〉 can be counted. Obviously, slight overlaps between clouds or unexpected transitions between eigenstates during the preparation of the initial state and/or the readout stage give errors and reduce the relevant fidelity values. These are SPAM errors related to our specific measurement system^38^.

Assuming that SPAM errors are mostly related to the readout imperfection, which can then be corrected, we perform a preliminary readout correction of the raw data. We prepare the state in |*j*〉 (*j*=0, 1 and 2), followed by an immediate qutrit readout for recording the probability value of correctly measuring the state in |*j*〉 and the other two probability values of incorrectly measuring the state in |*k*〉 (*k*≠*j*). The resulting 9 probability values can be used to construct the readout correction matrix. We note that this method may not be accurate since the state preparation error, though likely small, is ignored in constructing the correction matrix. However, the corrected experimental data agree well with the estimation from the full calibration of the STIRAP fidelity via concatenated pulses, as detailed in Supplementary Note 1, and with the calculated results using the master equation.

### Numerical simulations

We numerically calculate the level populations *P*_0_(*t*)=*ρ*_00_(*t*), *P*_1_(*t*)=*ρ*_11_(*t*), and *P*_2_(*t*)=*ρ*_22_(*t*) at any given time by solving the master equation


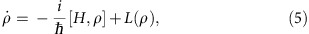


where *ρ* is the system's 3 × 3 density matrix, *H* is the Hamiltonian given by equation (1), and *L*(*ρ*) is the Liouvillean containing various relaxation and dephasing processes. Considering the general situation that the pump and Stokes microwaves are not correlated, we introduce a phase difference *ϕ* between the two microwaves in the actual calculations^39^. In this case, the double-rotating reference frame is described by the operator 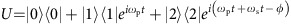, and the rotating-wave approximation leads to a Hamiltonian in the following form:


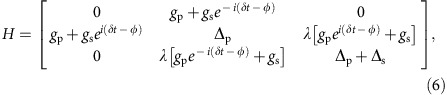


where the Liouvillean operator in equation (5) is given by^27^:


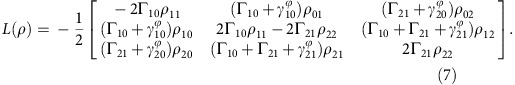


In our calculations *ρ*(*t*, *ϕ*) is obtained by solving equation (5) using the fourth-order Runge–Kutta method. When the phase difference *ϕ* of the two microwaves in our experiment is random, we average the result over *ϕ* and finally arrive at:


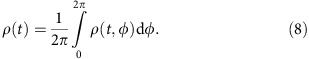


For the Xmon qutrit we use the parameters Γ_10_=8.4 × 10^4^ s^−1^, Γ_21_=1.3 × 10^5^ s^−1^, and 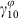
=2.0 × 10^5^ s^−1^ measured directly from experiment, and we estimate 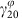
≈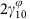 and 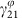
≈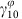 as in the case of phase qutrit (Supplementary Note 2).

## Additional information

**How to cite this article:** Xu, H. K. *et al*. Coherent population transfer between uncoupled or weakly coupled states in ladder-type superconducting qutrits. *Nat. Commun.* 7:11018 doi: 10.1038/ncomms11018 (2016).

## Supplementary Material

Supplementary Information Supplementary Figures 1-7, Supplementary Table 1, Supplementary Notes 1-2 and Supplementary References
