# Supplementary material for: Coherent population transfer between uncoupled or weakly coupled states in ladder-type superconducting qutrits
Source: Nat Commun. 2016 Mar 24;7:11018. doi: 10.1038/ncomms11018 (PMC4820826; doi:10.1038/ncomms11018)
Supplement: Supplementary Information — Supplementary Figures 1-7, Supplementary Table 1, Supplementary Notes 1-2 and Supplementary References [file ncomms11018-s1.pdf]

SUPPLEMENTARY FIGURES

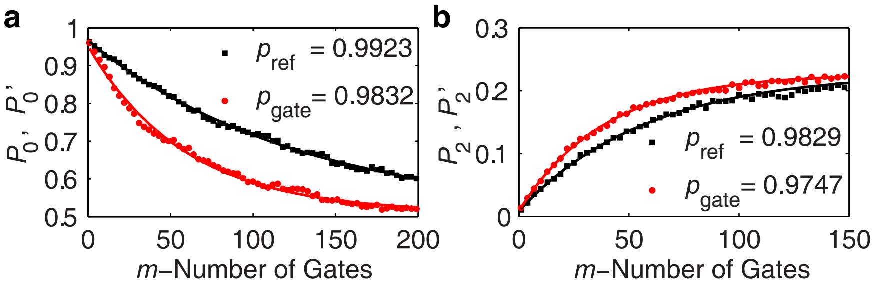

Supplementary Figure 1: Randomized benchmarking to calibrate the fidelity of X_{10} (**a**) and X_{21} (**b**). The x-axis is the number of Clifford gates m ($\leq N$) and the y-axis is the occupation probabilities of $|0\rangle$ in **a** and of $|2\rangle$ in **b** averaged over the K sequences. Probability values change exponentially with m due to the randomized accumulation of gate-specific errors. Dots are experimental data and lines are fits. In **b**, due to the state leakage to $|0\rangle$ caused by energy relaxation, we only fit the first part of the experimental data to keep the total sequence length within one third of the $|1\rangle$-state T_1 ($m \leq 60$ for red points and $m \leq 90$ for black points). The error rate of X_{10} (X_{21}) is estimated to be 0.46 % (0.42 %).

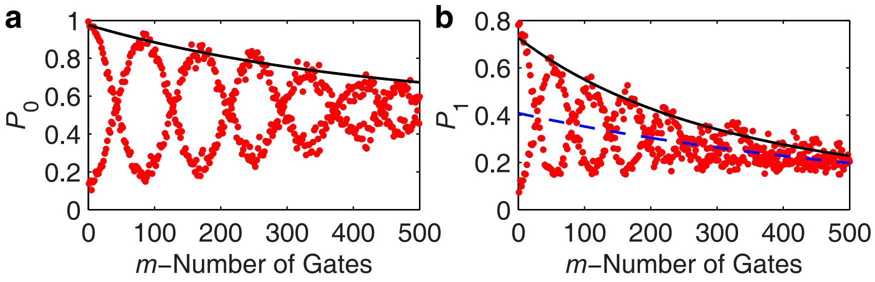

Supplementary Figure 2: Concatenating m-number of the same gates to calibrate the fidelity values of X_{10} (**a**) and X_{21} (**b**). The x-axis is the number of gates m and the y-axis is the occupation probabilities of $|0\rangle$ in **a** and of $|1\rangle$ in **b**. Envelopes change exponentially with m due to the accumulation of errors over the sequence length. Dots are experimental data and black solid lines are fits to the upper envelopes. In **b**, the blue dash line indicates a net decay of P_1 over a large m-number of gates. Due to the state leakage to $|0\rangle$ caused by energy relaxation, we take the average of the neighboring two probability values as B during the fit to Supplementary Equation (1). The gate fidelity p_{gate} of X_{10} (X_{21}) is estimated to be 99.77 % (99.53 %), and the error rate, $1 - p_{\text{gate}}$, of X_{10} (X_{21}) is therefore 0.23 % (0.47 %).

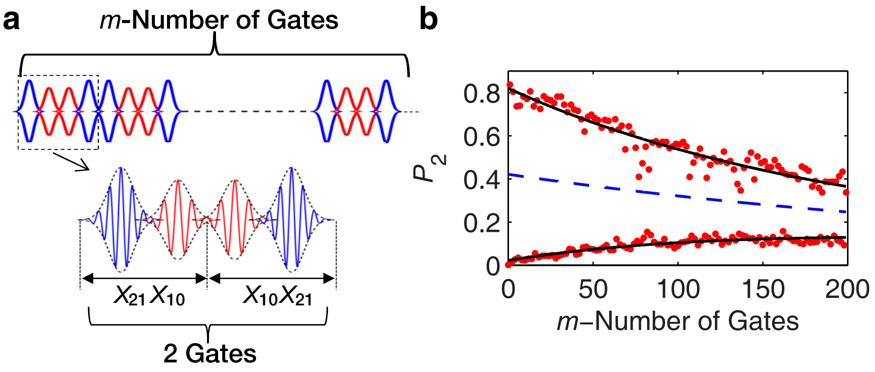

Supplementary Figure 3: Concatenating m-number of gates, $X_{21}X_{10}$ and $X_{10}X_{21}$, to calibrate their gate fidelities. (**a**) Top: pulse sequence consisting of m-Number of gates, with each gate combining two Gaussian pulses (the full width of each Gaussian pulse is 20 ns) with different tones indicated by their colors. Red corresponds to a tone for the $|1\rangle \leftrightarrow |2\rangle$ transition and blue corresponds to that for the $|0\rangle \leftrightarrow |1\rangle$ transition. Bottom: zoomed in view of two successive gates, $X_{21}X_{10}$ and $X_{10}X_{21}$. (**b**) The $|2\rangle$-state occupation probability, P_2, as function of the number of gates, m. Envelopes change exponentially with m due to the accumulation of errors over the sequence length. Dots are experimental data and solid lines are fits. Blue dash line indicates the effect due to state leakage to $|1\rangle$ caused by energy relaxation, which is obtained similarly as that in Supplementary Figure 2. Gate fidelities of both $X_{21}X_{10}$ and $X_{10}X_{21}$ are estimated to be 99.39 %, and the error rates, $1 - p_{\mathrm{gate}}$, of both $X_{21}X_{10}$ and $X_{10}X_{21}$ are therefore 0.61 %.

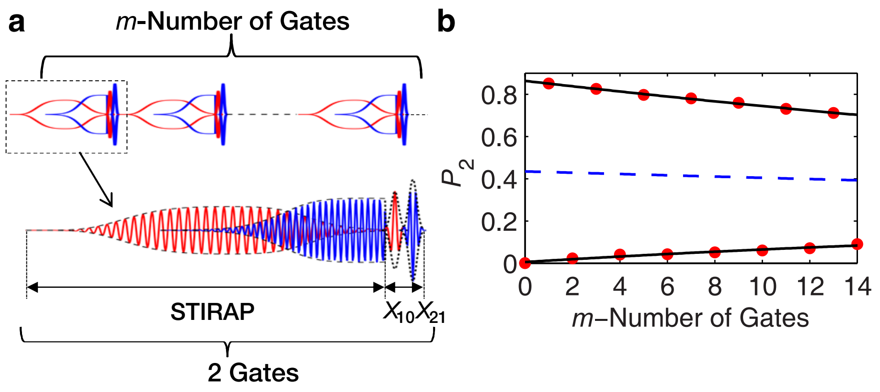

Supplementary Figure 4: Concatenating m-number of gates, STIRAP and $X_{10}X_{21}$, to calibrate the STIRAP fidelity. (**a**) Top: pulse sequence consisting of m-Number of gates, with each gate being either STIRAP or $X_{10}X_{21}$. Color indicates the tone for each pulse, as explained in Supplementary Figure 3 caption. Bottom: zoomed in view of two successive gates, STIRAP and $X_{10}X_{21}$. Sequences are drawn to scale, with the full width of the STIRAP pulse being 400 ns and that of $X_{10}X_{21}$ being 40 ns. (**b**) The $|2\rangle$-state occupation probability, P_2, as function of the number of gates, m. Envelopes change exponentially with m due to the accumulation of errors over the sequence length. Dots are experimental data and solid lines are fits. Blue dash line indicates the effect due to state leakage to $|1\rangle$ caused by energy relaxation, which is obtained similarly as that in Supplementary Figure 2. Taking the gate fidelity of $X_{10}X_{21}$ as 99.39 % from Supplementary Figure 3, The STIRAP fidelity is estimated to be, on average, 96.02 %, and the STIRAP error rate is therefore 3.98 %.

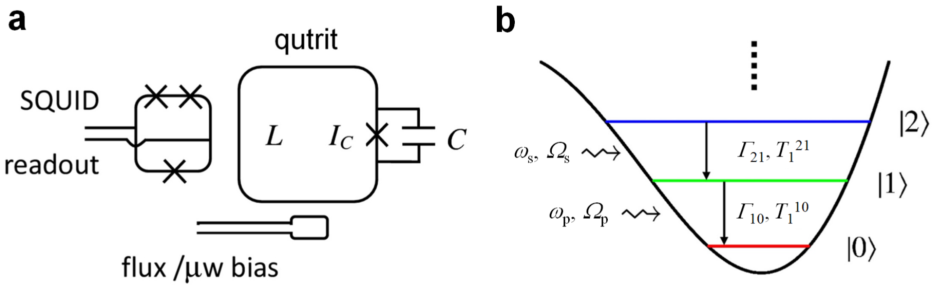

Supplementary Figure 5: Superconducting phase qutrit. (**a**) Schematic rf-SQUID type phase qutrit with Josephson critical current I_c, shunt capacitance C, and loop inductance L. (**b**) Three bottom energy levels $|0\rangle$, $|1\rangle$, and $|2\rangle$ of the qutrit with related symbols indicated. Subscripts p and s refer to the pump and Stokes tones, respectively.

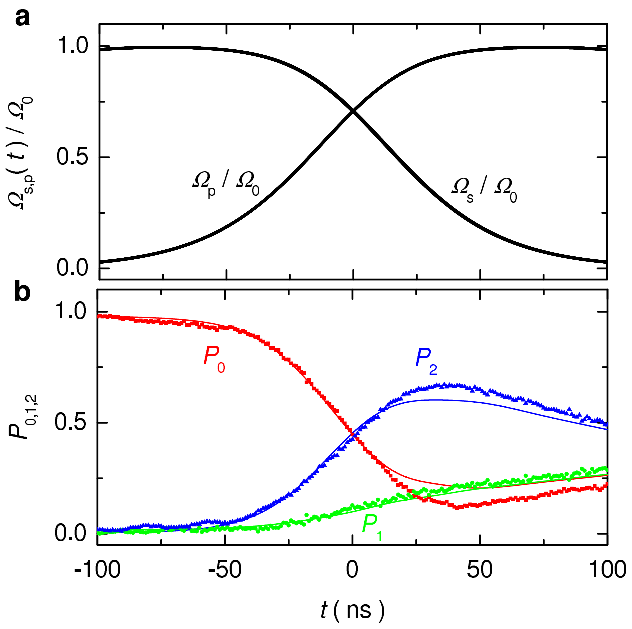

Supplementary Figure 6: Coherent population transfer via STIRAP in the superconducting phase qutrit. (**a**) Stokes and pump microwave pulses $\Omega_s(t)$ and $\Omega_p(t)$ shown in the overlapped region with the experimental parameters $\Omega_0/2\pi = 43$ MHz and $T_d = 100$ ns. (**b**) Level populations P_0, P_1, and P_2 versus time in the case of $\Delta_p = \Delta_s = 0$. A maximum experimental value of $P_2 = 67\%$, limited by the low coherence times of the device, is reached. Experimental and calculated results are shown as symbols and lines, respectively. The experimentally determined parameters listed in Supplementary Table 1 are used in the calculation.

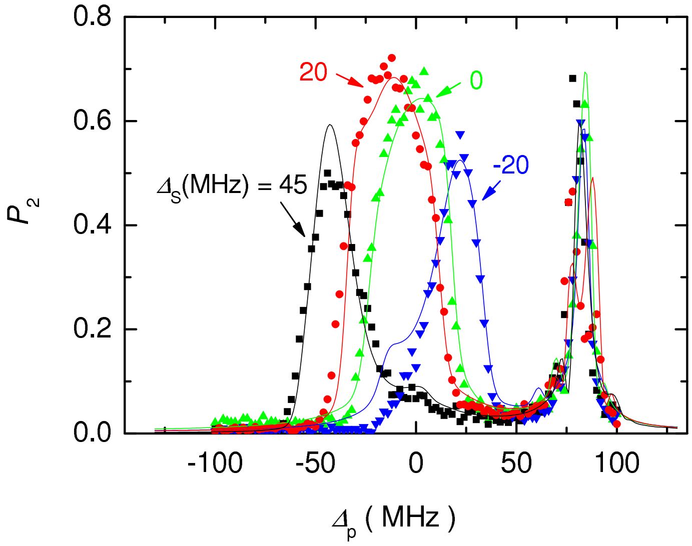

Supplementary Figure 7: Level population P_2 versus pump microwave detuning Δ_{p} for four Stokes tone detunings. Experimental and calculated results are shown as symbols and lines, respectively. The experimentally determined parameters listed in Supplementary Table 1 are used in the calculation. Bright resonances can be seen as the resonant condition is satisfied (left-side peaks in each curves). A maximum value of $P_2 = 72\%$ is reached at the microwave detunings $\Delta_{\mathrm{p}} = -\Delta_{\mathrm{s}} = -20$ MHz. The right-side peaks result from the two-photon process of the single pump microwave tone.

SUPPLEMENTARY TABLES

Supplementary Table 1: Parameters for the superconducting phase qutrit used in the present work.

Parameter	$\omega_{i,i-1}/2\pi$ (GHz)	$\Gamma_{i,i-1}$ (sec^{-1})	$\gamma^{\varphi}_{i,0}$ (sec^{-1})
$i = 1$	5.555	2.8×10^{6}	8.1×10^{6}
$i = 2$	5.393	5.1×10^{6}	$\sim 16.2\times10^{6}$

SUPPLEMENTARY NOTES

Supplementary Note 1: Calibration of STIRAP fidelity in Xmon qutrit

In our STIRAP experiment, the measured $|2\rangle$-state probability, P_2, is limited by both the STIRAP process errors and the state preparation and measurement (SPAM) errors, the latter of which are found to be dominant. In order to know the STIRAP fidelity, we can interleave the STIRAP process, which drives $|0\rangle \rightarrow |2\rangle$, and its reversal process, which effectively drives $|2\rangle \rightarrow |0\rangle$. By scaling up the number of concatenated STIRAP and its reversal processes the error rate of a single STIRAP process and its reversal can be estimated.

Here we first take a different approach to estimate the STIRAP fidelity, *i.e.*, comparing STIRAP with two successive π rotation gates, X_{10} and X_{21}, for driving $|0\rangle \rightarrow |1\rangle \rightarrow |2\rangle$. By optimizing the two rotation gates and their separation in time, we obtain P_2 values as high as 0.864 ± 0.011, in comparison with 0.852 ± 0.008 in STIRAP (see below). It is therefore concluded that the STIRAP fidelity should be comparable with the combined gate fidelity of $X_{21}X_{10}$. Then we demonstrate that the STIRAP fidelity can be directly calibrated by the method of concatenating gates for examining accumulated errors that scale with the pulse length.

A. Individual gate fidelities of X_{10} and X_{21}

Randomized benchmarking. We use two methods to calibrate the X_{10} (X_{21}) fidelity for the Xmon qutrit, both insensitive to SPAM errors. One is randomized benchmarking (RB) [1–3], and the other one simply concatenates X_{10} (X_{21}) for accumulating errors over the sequence length. The Xmon qutrit used in the experiment [4] has $T_1^{10} = 11.9\,\mu$s, $T_\varphi^{10} = 5.0\,\mu$s, and an anharmonicity $(\omega_{10}-\omega_{21})/2\pi$ = 227 MHz at $\omega_{10}/2\pi = 6.101$ GHz. The X_{10} (X_{21}) pulse is 20 ns in length, featuring a Gaussian envelope and a quadrature correction [5]. We use a dispersive readout scheme with additional gains from a parametric amplifier to detect the qutrit state.

In the RB method, the single-qubit Clifford group contains 24 rotations, which are decomposed into rotations around the x-axis or y-axis only [5]. We first numerically generate K sequences, each consisting of N random Clifford gates C_{ij}, where $i = 1, 2, \cdots, K$ and $j = 1, 2, \cdots, N$. Then we truncate the first m ($\leq N$) gates in each of the K sequences and interleave the m gates with X_{10}. A recovery Clifford gate $C_{r,i}$ is appended at the end such that $\prod_{j=1}^{m} C_{r,i}X_{10}C_{i,m-j+1} = I$. In the

experiment each sequence i of the K sequences is applied to the qutrit and the resulting qutrit $|0\rangle$-state probability, $P_{0,i}$, is measured. The averaged probability of the K sequences, $P_0 = \sum_{i=1}^{K} P_{0,i}/K$, decays exponentially with m due to randomized accumulation of gate-specific errors, *i.e.*, $P_0 = Ap_{\rm gate}^m + B$, where the gate error is captured by $p_{\rm gate}$ and the state preparation and measurement errors are captured by A and B (no dependence on m). To quantify the X_{10} error, we need to subtract from $p_{\rm gate}$ the error caused by imperfections in $\{C_{ij}\}$, denoted here as the error per reference, $p_{\rm ref}$. This is done by performing the experiment again with all the interleaved X_{10} gates removed in the sequences. Note that the final recovery Pauli gate $C_{r,i}$ should be changed accordingly such that $\prod_{j=1}^{m} C_{r,i}C_{i,m-j+1} = I$. The resulting P_0' versus m is fitted according to $P_0' = A' p_{\rm ref}^m + B'$. The error rate [3] of X_{10} is therefore $r_{\rm gate} = (1 - p_{\rm gate}/p_{\rm ref})/2$. Supplementary Figure 1a shows the experimental result with $K = 50$ and $N = 200$, suggesting an X_{10} error of less than 0.46 %.

RB for X_{21} is more subtle though the practice can be very similar. Here the Clifford gates are chosen from the subspace spanned by $|1\rangle$ and $|2\rangle$ of the Xmon system. The Xmon system is initialized in $|1\rangle$ before applying the RB sequences and the resulting averaged $|2\rangle$-state probability, P_2, versus m is used to extract the gate error. However, unlike the RB for X_{10}, there is the state leakage to $|0\rangle$ caused by energy relaxation for X_{21} case. To minimize the inaccuracy of the RB method, we fit the first part of the P_2 versus m data (numerically we find that using the $|2\rangle$-state probability instead of the $|1\rangle$-state one gives slightly larger errors and we limit the total sequence length to be around one third of the $|1\rangle$-state T_1 during fits). The experimental result with $K = 50$ and $N = 150$ is shown in Supplementary Figure 1b, where the error rate of X_{21} is estimated to be 0.42%. We emphasize again that the RB method for X_{21} may not be accurate due to the state leakage.

Concatenating gates In the other method, we apply m-number of X_{10} (X_{21}) gates sequentially to the qutrit, originally in $|0\rangle$ ($|1\rangle$), and then measure the $|0\rangle$-state ($|1\rangle$-state) probability P_0 (P_1). For a perfect X_{10} (X_{21}), we have $P_j = A(-1)^m + B$ for $j = 0$ (1), where A and B are related to SPAM errors. For a non-perfect gate with the gate fidelity $p_{\rm gate}$, accumulation of errors over the total m gates gives

$$P_j = A(-p_{\rm gate})^m + B, \tag{1}$$

for $j = 0$ (1), which can be used to estimate $p_{\rm gate}$.

During the experiment, we deliberately over-rotate by a small angle α in addition to the π rotation of X_{10} (X_{21}), so that each gate operator can be described by $\exp\left[-i(\tilde{\pi} + \alpha)/2\sigma_x\right]$. Here the symbol $\tilde{\pi}$ includes the rotation error. After m gates we have that $P_j = A(-p_{\rm gate})^m \cos(m\alpha) + B$ for

$j = 0$ (1). The resulted P_j versus m curve, $j = 1$ (2), which is oscillating due to the finite α, is used to estimate $p_{\rm gate}$.

Supplementary Figure 2a shows the data of P_0 versus m (red dots), together with a fit to the upper envelope (the black solid line), for concatenated X_{10} gates. Through the fit we estimate that $p_{\rm gate} \approx 99.77$ % and the error rate $1 - p_{\rm gate} \approx 0.23$ % for X_{10}, which is consistent with that from RB. Supplementary Figure 2b shows the data of P_1 versus m (red dots), together with a fit to the upper envelope (the black solid line), for concatenated X_{21} gates. Due to the state leakage to $|0\rangle$, we observe a net decay of P_1 over a large m-number of gates, indicated by the blue dash line as obtained by averaging the neighboring two probability values. If we take the blue dash line as B in Supplementary Equation (1), we estimate that $p_{\rm gate} \approx 99.53$ % and the error rate $1 - p_{\rm gate} \approx 0.47$ % for X_{21}, which is also consistent with that from RB. We note that fits to lower envelopes in Supplementary Figure 2a and 2b yield similar gate errors (data not shown).

B. Gate fidelity of $X_{21}X_{10}$

Previously we have estimated, via two methods, that individual gate fidelities of X_{10} and X_{21} are both above 99.50 %. As such the combined gate $X_{21}X_{10}$ for transferring $|0\rangle$ to $|2\rangle$ would yield an effective gate fidelity of above 99 %. We experimentally concatenate m-number of gates of $X_{21}X_{10}$ and its reverse, $X_{10}X_{21}$, for moving the qutrit state between $|0\rangle$ and $|2\rangle$ and then measure the accumulated errors.

Supplementary Figure 3 shows the pulse sequence and the resulting $|2\rangle$-state occupation probability. From the decayed envelope we estimated that the gate fidelities of both $X_{21}X_{10}$ and $X_{10}X_{21}$ are 99.39 %, which is in good agreement with the individual gate fidelity values of X_{10} and X_{21} obtained using the same method by concatenating gates.

C. Gate fidelity of STIRAP

One step further, we can concatenate m-number of gates, STIRAP and $X_{10}X_{21}$, which also effectively move the qutrit state between $|0\rangle$ and $|2\rangle$. The pulse sequence and the resulting $|2\rangle$-state occupation probability, P_2, are shown in Supplementary Figure 4, from which we can estimate that the STIRAP fidelity is, on average, 96.02 %. It is noted that we can only concatenate up to seven STIRAP pulses since each STIRAP pulse is 400 ns long.

Supplementary Note 2: STIRAP in phase qutrit

A. Sample parameters

In our work, coherent population transfer from state $|0\rangle$ to state $|2\rangle$ via the STIRAP process is also demonstrated in an Al-junction-based rf-SQUID type phase qutrit [6], which is shown schematically in Supplementary Figure 5. The sample is mounted on an oxygen-free copper platform thermally anchored to the mixing chamber of an Oxford cryogen-free dilution refrigerator and is cooled to $T \approx 10$ mK. The qutrit control and measurement circuit includes various filtering, attenuation, and amplification [7]. In the experiment, we bias the rf-SQUID to have six energy levels in the upper potential well and use the lowest three levels as the qutrit states. The relevant transition frequencies are $f_{10} = \omega_{10}/2\pi = 5.555$ GHz and $f_{21} = \omega_{21}/2\pi = 5.393$ GHz, and the relative anharmonicity is $\alpha = (f_{10} - f_{21})/f_{10} \approx 2.9\%$. The measured energy relaxation times are $T_1^{10} = 1/\Gamma_{10} = 353$ ns and $T_1^{21} = 1/\Gamma_{21} = 196$ ns, respectively, while the dephasing time determined from Ramsey interference experiment is T_φ^{10}=$1/\gamma_{10}^\varphi$=124 ns. We note that the low-frequency flux noise induced dephasing rate γ_{j0}^φ is proportional to $d\omega_{j0}(\Phi)/d\Phi$ ($j = 1, 2$) where Φ is the flux bias. Since the proportionality constant is determined by the integrated flux noise, which is the same for all values of j, and can be estimated from the measured γ_{10}^φ and $d\omega_{10}(\Phi)/d\Phi$, we are able to estimate γ_{20}^φ (and also γ_{21}^φ) from the measured $d\omega_{20}(\Phi)/d\Phi$ and the known proportionality constant. These parameters, which are used in our numerical simulations, are summarized in Supplementary Table 1.

B. Determination of level populations

The phase qutrit level populations $P_i(t)$ ($i = 0, 1, 2$) at a given time t are determined using two carefully calibrated nanosecond-scale measurement flux pulses A and B, which reduce the potential barrier to two different levels so that tunneling probabilities p^{A} and p^{B} in each case are measured. Pulse A leads to low tunneling probability p_0^{A}($\sim 5\%$) for state $|0\rangle$, high tunneling probability p_1^{A} for state $|1\rangle$ and, of course, even higher tunneling probability p_2^{A} for state $|2\rangle$. Pulse B results in a slightly deeper potential well than pulse A does so that $p_0^{\mathrm{B}} \simeq 0$, $p_1^{\mathrm{B}} \approx 5\%$, and a

much larger p_2^{B} for state $|0\rangle$, $|1\rangle$, and $|2\rangle$ respectively. Denoting the density operator of the qutrit as ρ, we have

$$p^{\mathrm{A,B}} = P_0 p_0^{\mathrm{A,B}} + P_1 p_1^{\mathrm{A,B}} + P_2 p_2^{\mathrm{A,B}} \ , \tag{2}$$

where $P_i = \rho_{ii}$, and $p_i^{\mathrm{A,B}}$ can be found [8] from the experimentally determined tunneling probabilities p_i of the ith energy level for given amplitudes of pulses A and B. Combining the normalization condition

$$\mathrm{T}r\rho = P_0 + P_1 + P_2 = 1 \ , \tag{3}$$

we obtain

$$P_i = [(p_j^{\mathrm{B}} - p_k^{\mathrm{B}})p^{\mathrm{A}} + (p_k^{\mathrm{A}} - p_j^{\mathrm{A}})p^{\mathrm{B}} + p_j^{\mathrm{A}} p_k^{\mathrm{B}} - p_k^{\mathrm{A}} p_j^{\mathrm{B}}] \ /D \ , \tag{4}$$

where $i = 0$, 1, 2 with $\{i, j, k\}$ in circulative order like $\{0, 1, 2\}$, $\{1, 2, 0\}$, and $\{2, 0, 1\}$, and D is the determinant

$$D = \begin{vmatrix} 1 & 1 & 1 \\ p_0^{\mathrm{A}} & p_1^{\mathrm{A}} & p_2^{\mathrm{A}} \\ p_0^{\mathrm{B}} & p_1^{\mathrm{B}} & p_2^{\mathrm{B}} \end{vmatrix} \ . \tag{5}$$

Hence the level populations P_0, P_1,and P_2 can be obtained by measuring p^{A} and p^{B}.

C. Coherent population transfer via STIRAP

Supplementary Figure 6a shows the two microwave pulses defined by $\Omega_0/2\pi = 43$ MHz and $T_d = 100$ ns in their overlapping region. The experimentally measured populations P_0, P_1, and P_2 versus time produced by the STIRAP pulse sequence in the resonant case $\varDelta_{\mathrm{p}} = \varDelta_{\mathrm{s}} = 0$ are plotted in Supplementary Figure 6b as symbols. The experimentally achieved maximum P_2, or the population transfer efficiency, is about 67% for the present sample under the resonant condition. The experimental results are reproduced well by the numerical simulation (solid lines) via the master equation using the experimentally determined parameters listed in Supplementary Table 1.

To check the conditions for the STIRAP process we note: $\delta/2\pi$ in the resonant case $\varDelta_p = \varDelta_s = 0$ is $f_{10} - f_{21} = 162$ MHz, which is approximately four times that of $\varOmega_0/2\pi$, and it is easy to verify that the integrated pulse area $\int_{-\infty}^{\infty} \sqrt{\varOmega_p^2(t) + \varOmega_s^2(t)}\, dt \approx 32\pi$ is greater than 10π.

In Supplementary Figure 7, we show the level $|2\rangle$ population P_2 versus the pump detuning $\varDelta_p$ for four Stokes tone detunings of $\varDelta_s = 45, 20, 0$, and -20 MHz, respectively. Experimental and calculated results are shown as symbols and lines. Bright resonance appears as the left-side peak in each curve when the two-photon resonant condition is met. It is seen that the maximum value of $P_2 = 72\%$ is reached at the microwave detunings of $\varDelta_p = -\varDelta_s = -20$ MHz, which is slightly higher than the value achieved in the resonant case of $\varDelta_p = \varDelta_s = 0$ shown in Supplementary Figure 6b. From the numerical simulations, maximum P_2 would appear at finite detunings when the qutrit relative anharmonicity is small. It is not observed for the Xmon device with a larger $\alpha = 3.7\%$ as described in the main paper. Such shift in the maximum efficiency vs detunings is possibly caused by the Stark shift [9,10] which is more effective in the case of small anharmonicity. In Supplementary Figure 7, the right-side peak in each curve is originated from the two-photon process excited by the single pump microwave tone. Compared to the left-side peaks, although the peak heights are comparable, they are much narrower, indicating that in practice it is less controllable using the two-photon process to perform coherent population transfer from state $|0\rangle$ to state $|2\rangle$.

SUPPLEMENTARY REFERENCES

1. Magesan, E., Gambetta, J. & Emerson, J. M. Scalable and robust randomized benchmarking of quantum processes. *Phys. Rev. Lett.* **106**, 180504 (2011).

2. Córcoles, A. D., Gambetta, J. M., Chow, J. M., Smolin, J. A., Ware, M., Strand, J., Plourde, B. L. T. & Steffen, M. Process verification of two-qubit quantum gates by randomized benchmarking. *Phys. Rev. A* **87**, 030301 (2013).

3. Kelly, J., Barends, R., Campbell, B., Chen, Y., Chen, Z., Chiaro, B., Dunsworth, A., Fowler, A. G., Hoi, I.-C., Jeffrey, E., Megrant, A., Mutus, J., Neill, C., O'Malley, P. J. J., Quintana, C., Roushan, P., Sank, D., Vainsencher, A., Wenner, J., White, T. C., Cleland, A. N. & Martinis, J. M.

Optimal quantum control using randomized benchmarking. *Phys. Rev. Lett.* **112**, 240504 (2014).

4. Sank, D., Barends, R., Bialczak, R. C., Chen, Y., Kelly, J., Lenander, M., Lucero, E., Mariantoni, M., Megrant, A., Neeley, M., O'Malley, P. J. J., Vainsencher, A., Wang, H., Wenner, J., White, T. C., Yamamoto, T., Yin, Y., Cleland, A. N. & Martinis, J. M. Flux noise probed with real time qubit tomography in a Josephson phase qubit. *Phys. Rev. Lett.* **109**, 067001 (2012).

5. Kelly, J. Fault-tolerant superconducting qubits. PhD thesis, University of California, Santa Barbara (2015).

6. Simmonds, R. W., Lang, K. M., Hite, D. A., Nam, S., Pappas, D. P. & Martinis, J. M. Decoherence in Josephson phase qubits from junction resonators. *Phys. Rev. Lett.* **93**, 077003 (2004).

7. Tian, Y., Yu, H. F., Deng, H., Xue, G. M., Liu, D. T., Ren, Y. F., Chen, G. H., Zheng, D. N., Jing, X. N., Lu, L., Zhao, S. P. & Han, S. A cryogen-free dilution refrigerator based Josephson qubit measurement system. *Rev. Sci. Instrum.* **83**, 033907 (2012).

8. Shalibo, Y., Resh, R., Fogel, O., Shwa, D., Bialczak, R., Martinis, J. M. & Katz, N. Direct Wigner tomography of a superconducting anharmonic oscillator. *Phys. Rev. Lett.* **110**, 100404 (2013).

9. Bergmann, K., Vitanov, N. V. & Shore, B. W. Perspective: Stimulated Raman adiabatic passage: The status after 25 years, *J. Chem. Phys.* **142**, 170901 (2015).

10. Di Stefano, P. G., Paladino, E., Pope, T. J. & Falci, G. Coherent manipulation of noise-protected superconducting artificial atoms in the Lambda scheme. Preprint at http://arXiv.org/quant-ph/1509.05562 (2015).
